# Evaluating the Information Content of Shallow Shotgun Metagenomics

**DOI:** 10.1128/mSystems.00069-18

**Published:** 2018-11-13

**Authors:** Benjamin Hillmann, Gabriel A. Al-Ghalith, Robin R. Shields-Cutler, Qiyun Zhu, Daryl M. Gohl, Kenneth B. Beckman, Rob Knight, Dan Knights

**Affiliations:** a Department of Computer Science and Engineering, University of Minnesota, Minneapolis, Minnesota, USA; b Bioinformatics and Computational Biology, University of Minnesota, Minneapolis, Minnesota, USA; c Biotechnology Institute, University of Minnesota, Minneapolis, Minnesota, USA; d Department of Pediatrics, University of California San Diego, San Diego, California, USA; e University of Minnesota Genomics Center, Minneapolis, Minnesota, USA; f Department of Computer of Science and Engineering, University of California San Diego, San Diego, California, USA; g Center for Microbiome Innovation, University of California San Diego, San Diego, California, USA; Duke University School of Medicine

**Keywords:** human microbiome, metagenomics, microbiome, shotgun metagenomics

## Abstract

Although microbial communities are associated with human, environmental, plant, and animal health, there exists no cost-effective method for precisely characterizing species and genes in such communities. While deep whole-metagenome shotgun (WMS) sequencing provides high taxonomic and functional resolution, it is often prohibitively expensive for large-scale studies. The prevailing alternative, 16S rRNA gene amplicon (16S) sequencing, often does not resolve taxonomy past the genus level and provides only moderately accurate predictions of the functional profile; thus, there is currently no widely accepted approach to affordable, high-resolution, taxonomic, and functional microbiome analysis. To address this technology gap, we evaluated the information content of shallow shotgun sequencing with as low as 0.5 million sequences per sample as an alternative to 16S sequencing for large human microbiome studies. We describe a library preparation protocol enabling shallow shotgun sequencing at approximately the same per-sample cost as 16S sequencing. We analyzed multiple real and simulated biological data sets, including two novel human stool samples with ultradeep sequencing of 2.5 billion sequences per sample, and found that shallow shotgun sequencing recovers more-accurate species-level taxonomic and functional profiles of the human microbiome than 16S sequencing. We discuss the inherent limitations of shallow shotgun sequencing and note that 16S sequencing remains a valuable and important method for taxonomic profiling of novel environments. Although deep WMS sequencing remains the gold standard for high-resolution microbiome analysis, we recommend that researchers consider shallow shotgun sequencing as a useful alternative to 16S sequencing for large-scale human microbiome research studies where WMS sequencing may be cost-prohibitive.

**IMPORTANCE** A common refrain in recent microbiome-related academic meetings is that the field needs to move away from broad taxonomic surveys using 16S sequencing and toward more powerful longitudinal studies using shotgun sequencing. However, performing deep shotgun sequencing in large longitudinal studies remains prohibitively expensive for all but the most well-funded research labs and consortia, which leads many researchers to choose 16S sequencing for large studies, followed by deep shotgun sequencing on a subset of targeted samples. Here, we show that shallow- or moderate-depth shotgun sequencing may be used by researchers to obtain species-level taxonomic and functional data at approximately the same cost as amplicon sequencing. While shallow shotgun sequencing is not intended to replace deep shotgun sequencing for strain-level characterization, we recommend that microbiome scientists consider using shallow shotgun sequencing instead of 16S sequencing for large-scale human microbiome studies.

## Abstract (precis)

A common refrain in recent microbiome-related academic meetings is that the field needs to move away from broad taxonomic surveys using 16S sequencing and toward more powerful longitudinal studies using shotgun sequencing. However, performing deep shotgun sequencing in large longitudinal studies remains prohibitively expensive for all but the most well-funded research labs and consortia, which leads many researchers to choose 16S sequencing for large studies, followed by deep shotgun sequencing on a subset of targeted samples. Here, we show that shallow- or moderate-depth shotgun sequencing may be used by researchers to obtain species-level taxonomic and functional data at approximately the same cost as amplicon sequencing. While shallow shotgun sequencing is not intended to replace deep shotgun sequencing for strain-level characterization, we recommend that microbiome scientists consider using shallow shotgun sequencing instead of 16S sequencing for large-scale human microbiome studies.

## INTRODUCTION

Despite the close association of microbial communities with many aspects of human, environmental, plant, and animal health (1–4), it is not currently possible to characterize precisely the species and genes present in a microbial community in a cost-effective manner. The microbial communities of human microbiomes are complex, multivariate, and multidimensional, requiring large studies to power novel biomarker discovery and predictive modeling (4, 5). Deep whole-metagenome shotgun (WMS) sequencing can provide highly resolved strain-level taxonomic and functional information but is generally cost-prohibitive for large-scale studies. Many of the largest microbiome studies to date have been performed via 16S rRNA gene amplicon (16S) sequencing, a cost-effective alternative, but 16S sequencing typically provides only genus-level taxonomic assignments (6) and rough estimates of the functional repertoire (7, 8), limiting the amount of information that can be learned from the data. The purpose of this paper is to evaluate shallow shotgun sequencing as a possible cost-effective alternative to 16S sequencing for large-scale biomarker discovery with improved taxonomic resolution and functional accuracy.

A major concern for the use of any microbiome assay is the ability to identify and quantify taxonomic and functional traits from within a complex community. Deep WMS sequencing has a number of advantages over 16S sequencing for microbiome profiling for these purposes in well-characterized environments; for example, deep WMS sequencing of mixed communities, such as the human gut microbiome, has been effective at recovering strain-level polymorphisms and functional traits for abundant strains (9–11). Both shallow and deep shotgun sequencing are also less subject to amplification bias than 16S sequencing because they do not rely on targeted primers to amplify a marker gene (12). However, at the time of writing, WMS sequencing typically costs several times more per sample than 16S sequencing for library preparation and DNA sequencing. In addition to that of the sequencing itself, the cost of WMS sequencing includes labor and reagents for quality control and sequencing library preparation.

Although extensive work has been done to characterize how many 16S sequencing reads are required for quantifying relevant biological signals of different types, the same has not been done for alpha diversity, beta diversity, species profiles, and functional profiles with shotgun metagenomics. One prior study estimated the average coverage of each microbe given its relative abundance in a metagenomic sample at a given depth, concluding that approximately ∼7 Gb of pairs of sequencing data were required to achieve >20× coverage of each microbe above 1% relative abundance (13). Another study compared 16S and WMS sequencing by rarefying reads from depths of 500 to 100,000 repeatedly, concluding that 16S sequencing has significant primer bias. This study mentioned that 16S sequencing is typically used for dense sampling efforts, including longitudinal studies (14). Another study explored the use of database-free methods for metagenomic analysis and found that approximately 1 Gb of pairs would provide sufficient metagenomic coverage to compare metagenomic samples with such methods (15).

The depth necessary for metagenomic sequencing in a particular study depends on the purpose of the study; in many studies, the key goals are to understand which species and functions are present and to identify biomarkers related to experimental groups or outcomes. To address the important question of how many reads are required to capture species-level taxonomic and functional assignments (e.g., KEGG Orthology groups [16]), we analyzed shotgun sequencing data at various depths. We found that shotgun sequencing can produce species and functional profiles at a level of quality similar to that of deep WMS sequencing using few as 0.5 million sequences per sample, as demonstrated on deep whole-genome sequencing (WMS) samples from the Human Microbiome Project (HMP) data set (4), the HMP mock community (12), a diabetes study (17), simulated human gut microbiomes, and two novel human stool samples on which we performed ultradeep sequencing of 2.5 billion sequences per sample.

## RESULTS

**FIG 1:**
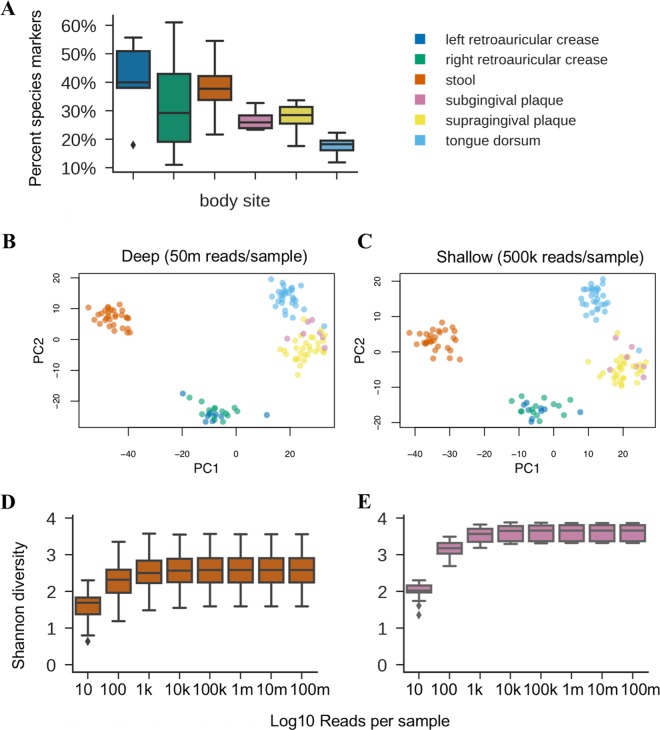
Information content of deep and shallow shotgun sequencing. (A) Percentages of raw shotgun DNA sequences that are unique to one bacterial species across different human body habitats (7 distinct plaque samples, 30 distinct samples from other body sites). (B, C) Principal-coordinate analysis of Bray-Curtis beta diversity using deep (B) and shallow (C) sequencing (sample sizes were as described for panel A). (D, E) Shannon diversity estimates at varied sequencing depths for human stool (D) and subgingival plaque microbiomes (E) (sample sizes were as described for panel B). Boxplots show minimums, first quartiles, medians, second quartiles, and maximums, with outliers beyond 1.5 times the interquartile range plotted individually.

**FIG 2:**
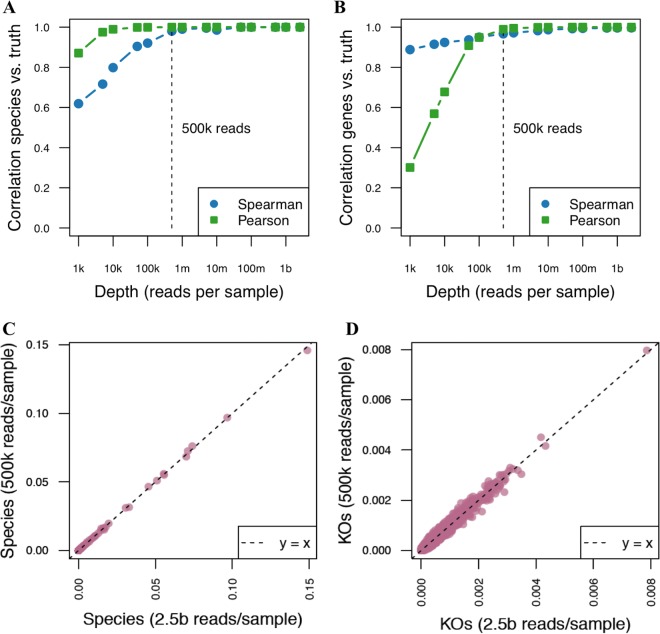
Comparison of species and function profiles with ultradeep sequencing data. (A, B) Correlation with ground truth species (A) and KEGG Orthology group (KO) (B) profile for known genes present in the reference database at different sequencing depths, showing that as few as 0.5 million sequences recover nearly the full species and function profiles (ground truth based on 2.5 billion reads per sample; 4,394 genes and 694 species were used at each subsampling level from the subject 1 ultradeep sequencing sample; comparable results from subject 2 are not shown). Gene and species profiles recovered from the ultradeep data include only direct matches to genes and genomes present in the database; *de novo* assembly of novel genes and contigs from deep data are expected to yield additional uncharacterized gene content and is not possible with shallow shotgun data. (C, D) Scatterplots of species (C) and KOs (D) at 0.5 million versus 2.5 billion reads per sample (we used the same sample size as used for panel A and B above).

**FIG 3:**
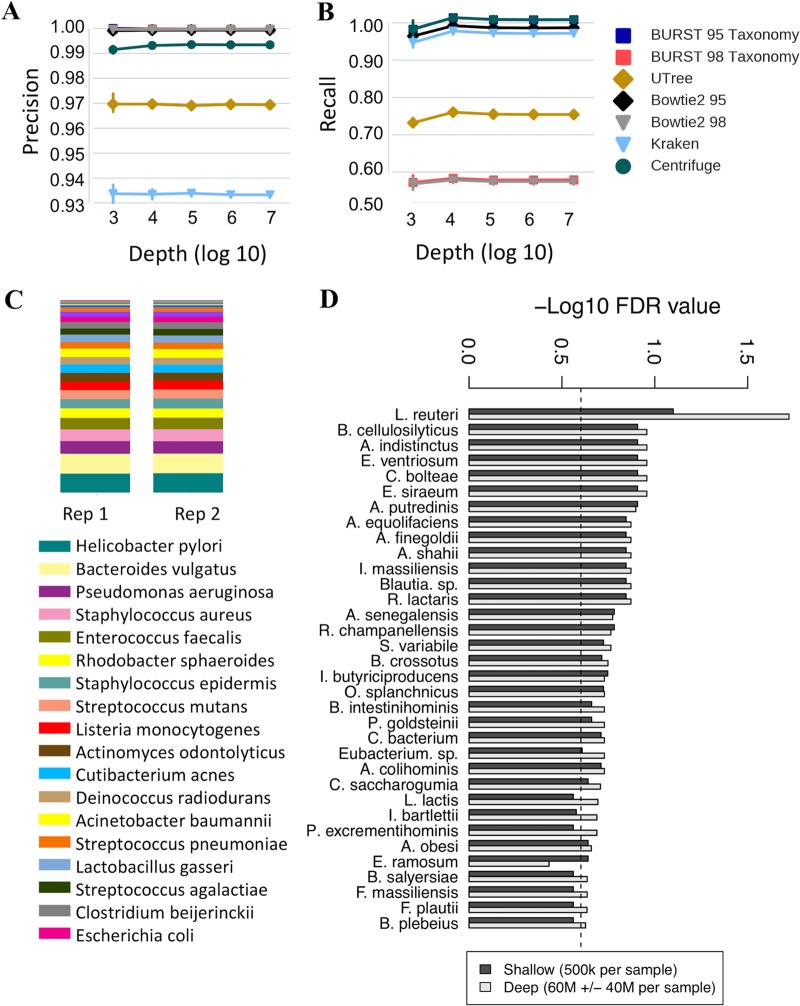
Biomarker discovery using shallow shotgun sequencing. (A, B) Precision, recall for per-read species binning of different metagenomics analysis tools (“95” and “98” refer to the minimum alignment identity threshold used; 5 distinct replicates [rep] were performed per subsampling depth, and error bars show standard deviations). (C) Stacked bar plot of species abundances recovered from HMP mock community shotgun sequencing data. (D) Negative log_10_ false-discovery rate (FDR)-corrected *P* values using Mann-Whitney U tests for species associated with type 2 diabetes (17), compared between deep and shallow shotgun sequencing (43 healthy patients, 53 patients with type 2 diabetes).

A comparison between deep WMS sequencing and shallow shotgun sequencing in real and simulated biological data sets demonstrated that shallow shotgun sequencing provides nearly the same accuracy at the species and functional level as deep WMS sequencing for known species and genes in five key aspects of microbiome analysis: (i) beta diversity (Fig. 1B and C); (ii) alpha diversity (Fig. 1E and F); (iii) species composition (Fig. 2A and C); (iv) functional composition (Fig. 2B and D); and (v) clinical biomarker discovery (Fig. 3).

### Alpha and beta diversity profiling.

We obtained deep WMS sequencing data from the Human Microbiome Project (HMP) (4) and subsampled the data to simulate shallow shotgun sequencing depth across five body subsites representing skin, oral, and gut habitat microbiomes. Surprisingly little sequencing was needed to recover the diversity trends found in deep WMS sequencing data. We annotated the deep WMS sequencing and the shallow shotgun sequencing data using an accelerated version of fully exhaustive gapped Needleman-Wunsch alignment (18, 19) for both taxonomic and functional profiles, against all complete representative bacterial genomes from the reference database RefSeq version number 82 (20). Fully exhaustive alignment allowed us to identify any and all ties for best match for each input sequence according to sequence identity. Species relative abundance profiles were derived by tabulating the number of sequences with at least 80% of the best hits belonging to one species. This is similar to the direct mapping approach used commonly in k-mer-based approaches to shotgun metagenomics taxonomic profiling (21) but with higher sensitivity and recall due to the use of gapped sequence alignment (see the simulated data analysis below). Using the fully exhaustive alignment approach, we found that 20% to 40% of all sequences could be identified as species markers because they were uniquely present in only one species in the database (Fig. 1A). We then bootstrapped these samples repeatedly down to 10, 100, 1,000, 10,000, 100,000, and 1 million sequences per sample and reran the analysis to quantify species-level alpha diversity and beta diversity profiles. In all cases, a depth of 0.5 million sequences was more than sufficient to recover the same alpha- and beta-diversity signals, as with deep WMS sequencing (Fig. 1B and D). We used Procrustes analysis (22) to confirm that the beta diversity matrices based on shallow and deep data were similar (Procrustes test, *P* value = 0.001).

### Species and functional profiles in human stool samples.

In order to compare the performances of shallow shotgun sequencing for species and functional profiling with ultradeep sequencing, we obtained ultra-deep WMS sequencing of 2.5 billion sequences per sample on novel human stool samples from two individuals. At the time of writing, to the best of our knowledge, this was the deepest sequencing that had been performed on human stool microbiomes. Using these two novel samples, we measured the species profiles and functional profiles as described above with exhaustive gapped alignment against a full genome database for species and a database of genes annotated with KEGG Orthology groups (KOs) (16). We performed this analysis at the full depth of 2.5 billion sequences and then subsampled to lower depths. Species profiles at 0.5 million sequences per sample had an average correlation of 0.990 with ultradeep WMS sequencing data (Spearman correlation, *n* = 112, *P* < 2 × 10^−16^) across the two samples (Fig. 2A and C), and the average KO profile correlation was 0.971 (Spearman correlation, *n* = 4,394, *P* < 2 × 10^−16^) (Fig. 2B and D). For KO annotation, we used direct gene observation for all but the lowest-abundance genes, for which we augmented the direct KO counts with counts of all KOs contained in observed reference strains in a manner similar to that of Piphillin (8). We weighted the amount of augmentation according to the coefficient of variation of a binomial distribution at the given observed proportion for a given gene, with the augmented gene counts contributing at most 10% of the total counts for a given gene. In practice, this approach affects only the rarest genes with fewer than approximately 10 direct observations and offers a slight improvement in accuracy in Spearman correlation with virtually no change to Pearson correlation (see Materials and Methods). Our observed 97.1% Spearman correlation of the shallow and deep functional profiles is substantially higher than the correlations of functional profiles predicted from 16S sequencing, which typically has 80% to 90% correlation with the directly observed functions (7, 8). We note that we are evaluating the correspondence of the gene profiles using the set of known genes present in the reference database. We did not assemble genes and genomic regions *de novo* from the deep shotgun data for comparison, as the lack of an ability to assemble new genes and genomes is a fundamental limitation of shallow shotgun sequencing (see Discussion).

Following the comparison of shallow shotgun sequencing to ultradeep sequencing of real biological samples, we also simulated deep WMS sequencing of complex metagenomes from a reference database to evaluate precision and recall of shotgun sequencing at different depths. Individual sequences were drawn at random from full reference genomes of selected species, with a simulated 5% rate of sequencing error. Three different mixtures of species were selected from the database to match the average species-level composition of HMP samples from stool, oral, and skin body sites, respectively (see Materials and Methods). Precision was defined as the fraction of simulated reads that were correctly assigned to their respective species divided by the total number of reads that mapped to the database. Recall was defined as the fraction of simulated reads that were correctly assigned to their respective species divided by the total number of simulated reads. We found similarly high precision rates of 0.985 to 0.995 when using exhaustive gapped alignment, Bowtie2 (23) at 95% or 98% alignment identity, or Centrifuge (24); k-mer-based methods, including Kraken (21) and an in-house method for comparison (25), had lower precision (Fig. 3A). Recall was considerably higher with 95% identity than with 98% identity alignment with exhaustive gapped alignment or Bowtie2, likely due to the high error rate in the simulated data (Fig. 3B). We also analyzed published shotgun data from the HMP mock community (12), recovering all expected species perfectly as the top 20 taxa, with the exception of Bacillus cereus which was recovered at the genus level due to highly overlapping species genomes in the genus *Bacillus* (26) (Fig. 3C).

### Species-level biomarker discovery.

To assess the ability of shallow shotgun sequencing to identify species-level biomarkers in a clinical study, we subsampled deep shotgun sequencing data from a study of stool microbiomes from healthy individuals and individuals with type 2 diabetes (T2D) (17) to 0.5 million sequences per sample. We identified the species significantly associated with T2D in both the deep data and the shallow data using two-sided Mann-Whitney U tests and found high concordance between the *P* values for species down to a 0.0005 relative abundance (rank correlation of lists of differentially abundant species were ordered by raw *P* value; rho = 0.954 across 10 subsampled replicates; *n* = 94; *P* < 2 × 10^−16^), indicating that 0.5 million sequences per sample enables discovery of species-level biomarkers with power comparable to deep that of shotgun sequencing down to a relative abundance of approximately 0.0005 (Fig. 3D). Notably, this classification task contained a range of statistical signals ranging from very strong to marginally significant.

### Comparison to 16S sequencing species profiles.

**FIG 4:**
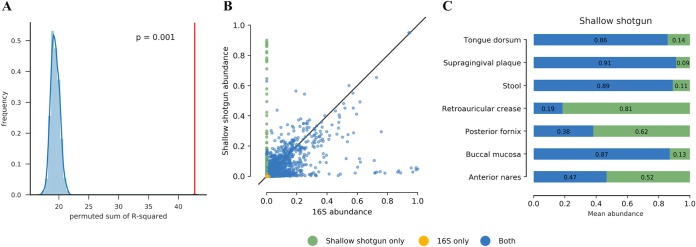
Comparison of 16S sequencing and shallow shotgun recovery of species-level taxa. (A) Histogram of average Pearson correlation (*R*-squared) of species profiles between 16S sequencing and shallow shotgun sequencing from the same HMP sample (*R*-squared = 0.918), compared to the permutation-based null distribution of *R*-squared values for random pairings (*P* < 0.001). (B) Scatterplot of relative abundances of species in shallow shotgun sequencing versus 16S sequencing from the same HMP samples. Species found in only one data type are shown in a different color. (C) Fractions of all observed species, with relative abundance accounted for by species found by 16S sequencing only, shallow shotgun sequencing only, or both.

As noted, 16S sequencing variable-region amplicon sequences often do not resolve taxa below the genus or family level, although some species can be differentiated with 16S sequencing (6). To compare the overall concordance between 16S sequencing species profiles and shallow shotgun sequencing species profiles for pairs from the same sample, we calculated the Pearson correlation *R*-squared value (coefficient of determination) of the 16S sequencing and shallow shotgun sequencing species profiles in paired samples from the HMP (see Materials and Methods; Table S1) and found that the average *R*-squared was 0.918. This demonstrated high overall concordance between 16S sequencing and shallow shotgun sequencing species profiles within a subject. We then permuted the pairing of the 16S sequencing and shallow shotgun sequencing profiles and repeated the average *R*-squared calculation to obtain a null distribution, showing that the *R*-squared value between the true pairs of samples was better in all cases than the randomly assigned pairs (Monte Carlo permutation test, *P* < 0.001) (Fig. 4A). To compare the contributions to total relative abundance of observed species between 16S sequencing and shallow shotgun sequencing profiles, we merged the species-level taxonomic profiles for paired 16S sequencing and shallow shotgun sequencing analyses and measured the fraction of species attributed to 16S sequencing only, shallow shotgun sequencing only, or both. We found that there were many species observed only in the shallow shotgun sequencing data, with some observed at high levels of abundance (Fig. 4B and C), indicating that the 16S sequencing identified a subset of the dominant taxa at the species level. A higher concordance was observed between genus-level profiles from 16S sequencing and shallow shotgun sequencing than between species-level profiles from 16S sequencing and shallow shotgun sequencing (Fig. S1). We confirmed that these results were similar when we compared the 16S sequencing data to deep shotgun metagenomics of the HMP data (Fig. S1).

10.1128/mSystems.00069-18.1 FIG S1 Comparison of deep shotgun, 16S sequencing, and shallow shotgun sequencing recovery for species-level and genus-level taxa. (A) Scatterplot of relative abundances of bacterial genera recovered from shallow shotgun sequencing versus 16S sequencing data. (B) Fractions of genera observed by 16S sequencing only, shallow shotgun sequencing only, or both. (C, D) Scatterplots showing concordance of genus-level (C) and species-level (D) taxonomy profiles in HMP samples between deep and shallow shotgun sequencing data. Download FIG S1, PDF file, 0.1 MB. Copyright © 2018 Hillmann et al. 2018 Hillmann et al. This content is distributed under the terms of the Creative Commons Attribution 4.0 International license.

10.1128/mSystems.00069-18.2 TABLE S1 Shotgun metagenomics Human Microbiome Project samples used in this analysis. Download Table S1, TXT file, 0.02 MB. Copyright © 2018 Hillmann et al. 2018 Hillmann et al. This content is distributed under the terms of the Creative Commons Attribution 4.0 International license.

## DISCUSSION

In this work, we evaluated the information content of shallow shotgun sequencing as a potential alternative to 16S sequencing in certain situations. We found that surprisingly few shotgun metagenomic sequences are needed to obtain more reliable species and gene group profiles than 16S sequencing at approximately the same cost as 16S sequencing. We also compared shallow shotgun sequencing to deep shotgun sequencing of a number of biological data sets, including samples from the HMP, a published deep shotgun diabetes study, and simulated and mock communities, and found that we could recover similar trends in alpha and beta diversity, species profiles, and species biomarker discovery down to a 0.05% relative abundance with as few as 0.5 million sequences per sample. We then analyzed two human stool samples with new ultradeep shotgun sequencing data at 2.5 billion reads per sample, the deepest sequencing coverage yet obtained of any microbiome to our knowledge. We found that shallow sequencing recovers 97% to 99% of the correlated species and KEGG (16) Orthology group (KO) profiles found by ultradeep sequencing. Although we obtained shallow shotgun sequencing on the data described in this paper by subsampling originally deep shotgun sequencing data, we expect subsampling to provide an unbiased representation of the data that would be observed in actual shallow shotgun sequencing. We also found, using HMP samples with paired 16S sequencing and shotgun data, that shallow shotgun sequencing is superior to 16S sequencing for recovery and annotation of species.

We did not attempt to perform an exhaustive comparison of different sequence annotation tools, as that was outside the scope of our investigation. Instead, we selected several tools representing different approaches to database search for comparison, including exhaustive semiglobal gapped alignment (18, 19), heuristic gapped alignment using the Burrows-Wheeler transformation (23), and k-mer-based searching (21, 25). Using simulated metagenomic data, we found that tools using gapped alignment obtained higher precision and recall than tools using k-mer-based mapping. This result was expected, as k-mer mapping requires exact matches of fixed-size k-mers, whereas gapped alignment allows insertion of gaps at random to maximize overall sequence identity. In our simulated data, fully exhaustive end-to-end gapped alignment with a minimum threshold of 95% identity using an accelerated version of Needleman-Wunsch alignment (18, 19) performed best in terms of recall. Several methods were approximately tied for highest precision. A potential advantage of gapped alignment over k-mer mapping is that current alignment-based tools report the genomic coordinates of each match, allowing estimation of strain-level coverage, which may be useful for future work into novel algorithms that use strain-level coverage to further improve precision and recall for rare species.

We note a number of important limitations to shallow shotgun sequencing. Shallow shotgun sequencing, as with deep shotgun sequencing, may not be a viable replacement for 16S sequencing when characterizing blood or biopsy specimen microbiomes, where there is likely to be more host DNA contamination and relatively low bacterial biomass. Shallow shotgun sequencing does not allow *de novo* assembly of genes and genomes and, thus, relies on whole-genome reference databases and will require expansion of reference genomes to cover novel environments. When analyzing poorly characterized environments, researchers may consider combining 16S sequencing for identification of novel taxonomic groups with shallow shotgun sequencing for functional profiling. We have not attempted to compare deep or shallow shotgun sequencing with 16S sequencing in environments with a low representation of strains in the reference database, such as marine or soil samples. In these cases, it is likely that shallow shotgun sequencing will still reveal useful functional profiles due to the homology of some observed sequences to known genes and species, but we expect 16S sequencing to provide superior profiling of novel taxa due to the lack of available representative genomes covering endemic species, as has been observed for freshwater samples (27).

Shallow shotgun sequencing is not meant to be a replacement for deep WMS sequencing for strain-level resolution or tracking polymorphisms in strains. Shallow shotgun sequencing cannot be used for novel gene and genome assembly. Our analysis has compared the performance of shallow shotgun sequencing metagenomics to those of 16S sequencing and deep shotgun metagenomics for known genes and genomes only; *de novo* assembly of genes and genomes directly from deep shotgun metagenomic data is likely to reveal novel gene content not readily identifiable from shallow shotgun sequencing data. For many of the metrics that we examined, a depth of 0.5 million sequences per sample was sufficient, but deeper sequencing is warranted for detection of rare species below a relative abundance of approximately 0.0005. We chose 0.5 million for evaluation here because it is the highest depth at which the sequencing cost is still less than approximately half of the total cost of generating data, and yet it is a depth at which one still obtains reasonable sensitivity for species-level recovery; however, we do encourage readers to increase depth toward 1 million or 2 million reads when budget allows, as this will continue to increase sensitivity for detecting rare species. Still, we found that depths of as low as 1,000 reads per sample may be sufficient for some purposes, such as assessing differences in alpha diversity and beta diversity. Therefore, the minimum depth required is dependent on the experimental hypothesis under consideration. In addition, a general concern with any taxonomic annotation from shotgun metagenomic data is that the boundaries of traditional species taxonomic labels do not necessarily reflect consistent entities at the genomic level when accounting for horizontal gene transfer and inaccurate database annotations. These concerns can be alleviated to some extent using deep shotgun sequencing and metagenomic assembly (10), coabundance clustering (11), or proximity-based assembly (28), and *de novo* identification of strains from complex metagenomes remains an active area of research.

We found that shallow sequencing of human stool microbiomes provides high-quality species and functional profiles of human microbiome samples for little more than the cost of 16S amplicon sequencing when a miniaturized library preparation protocol was used (see Materials and Methods). We have made available the gene and genome databases that we used together with a convenient Python-based wrapper script that allows users to compare several existing tools for performing both taxonomic and functional annotation (see Materials and Methods). Shallow shotgun sequencing has a number of important limitations and is not intended to replace deep whole-genome shotgun sequencing for strain-level analysis or novel gene and genome assembly. Nonetheless, shallow shotgun analysis provides considerably more accurate functional profiles and more precise taxonomic resolution than 16S amplicon sequencing for human microbiome studies. Thus, shallow shotgun sequencing is a viable alternative to 16S sequencing for researchers performing large-scale human microbiome studies where deep shotgun sequencing may not be possible.

## MATERIALS AND METHODS

### Alignment algorithms.

Alignment was performed using several existing tools and algorithms, including Bowtie2 (23), Centrifuge (24), Kraken (21), an in-house k-mer-based aligner for comparison with Kraken (25), and an accelerated adaptation of Needleman-Wunsch alignment for exhaustive gapped semiglobal alignment (18, 19).

### Shotgun species profiling.

After sequences were trimmed until the quality score was above 20 and trimmed sequences shorter than 80 bases or with average quality score of less than 30 were discarded, query reads were mapped with several different alignment tools against representative and reference genomes from the RefSeq database, version 82 (20), using a 95% identity threshold (also compared to 98% for precision and recall evaluation on the simulated data). A read that mapped to a single reference genome is labeled with the NCBI taxonomic annotation. All reads that mapped to multiple reference genomes are labeled as the last common ancestor (LCA) of each label according to the NCBI taxonomy, and only species-level assignments are retained. We use a confidence-adjusted LCA that requires at least 80% of all tied best matches to agree for species annotation. All source codes can be found at the GitHub repository (https://github.com/knights-lab/SHOGUN). Additional analysis code used to generate figures and run tests for this paper can also be found in GitHub (https://github.com/knights-lab/analysis_SHOGUN).

### Shotgun functional profiling.

Functional profiling was obtained using KEGG Orthology group (KO) (16) annotations for RefSeq-derived genes (20) from directly observed exhaustive gapped alignments in ultradeep WMS sequencing. To improve the accuracy of the direct KO profiles for low-abundance genes, the KO profiles were separately predicted from reference genomes and the predicted profiles were used to augment the estimates of low-abundance KOs. Specifically, we identified those query reads with a 100% match to exactly one reference genome and predicted the entire KO profile of that genome to be present in the sample, which is similar to a previously published approach (8). This is similar to the PICRUSt algorithm for amplicon sequencing data (7) but without the intermediate steps of clustering short-read amplicons and identifying closely related reference genomes. The final KO profiles reported by SHOGUN are a weighted average of predicted and directly observed KO profiles. The predicted KO counts are weighted between 0.0 and 0.1 by a linear function of the coefficient of variation of the count for a given KO, estimated from the size of the binomial confidence interval for the observed count of a given KO divided by the count of that KO. The direct KO profiles receive the remainder of the weight, such that the direct KO profiles receive at least 90% of the weight for all genes and the predicted KO profiles are trusted only for the lowest-abundance genes for which the expected variance in observed count is high. 16S sequences were aligned to Greengenes version 13_8 (29) at 98% identity with exhaustive gapped alignment (18, 19). Where a query sequence aligned equally well to multiple reference sequences, the taxonomic assignment was made using the last common ancestor conserved across at least 80% of the set of references.

### Human microbiome project data.

We obtained deep WMS sequencing data from the Human Microbiome Project (HMP) (4) and subsampled the data to simulated shallow shotgun sequencing depth. We annotated the deep WMS sequencing data using fully exhaustive gapped alignment for both taxonomy and functional profiles against all complete representative bacterial genomes from the reference database RefSeq version number 82 (20). We then rarefied these samples repeatedly to 1,000, 10,000, 100,000, 1 million, and 10 million sequences per sample and ran the SHOGUN pipeline to quantify species and gene profiles. The list of HMP WMS sequencing and corresponding 16S sequencing samples used are provided in Tables S1 and S2, respectively. The HMP mock community data are from runs SRR2726671 and SRR2726672 from NCBI accession number SRX1342165 (12).

10.1128/mSystems.00069-18.3 TABLE S2 16S Human Microbiome Project samples used in this analysis. Download Table S2, TXT file, 0.4 MB. Copyright © 2018 Hillmann et al. 2018 Hillmann et al. This content is distributed under the terms of the Creative Commons Attribution 4.0 International license.

### Simulated human metagenomes.

The body sites analyzed from the HMP1 project were first grouped according to the broad stool, skin, and oral body sites. We calculated the average relative abundances of all samples within each group. The 100 most abundant species for each group were used for simulating communities. The reads were simulated from a randomly selected strain belonging to each of those most abundant species according to the average proportion of that species in the respective body site group using the tool dWMSim (30). The reads were simulated with default settings for Illumina single-end sequencing machines with a 5% mutation rate where 2% of mutations are indels and with a maximum of 10 ambiguous bases per query sequence.

### Sequencing library preparation.

Shotgun DNA sequencing was performed on the Illumina HiSeq platform. DNA was extracted using the Qiagen DNeasy PowerSoil kit and was quantified using the Quant-iT PicoGreen dsDNA assay (Thermo Fisher). DNA sequencing libraries were prepared using one-quarter-scale NexteraXT reactions (Illumina). The resulting DNA libraries were denatured with NaOH, diluted to 8 pM in Illumina’s HT1 buffer, and spiked with 1% PhiX, and a HiSeq 1× 100-cycle v3 kit (Illumina) was used to sequence samples. For the ultradeep shotgun sequencing, 64 separate libraries were prepared as described above but using full Nextera reaction mixtures from a homogenized stool sample and were multiplexed on a HiSeq 3000 high-output run, using an entire run per sample.

### Ethics statement.

Volunteers contributing stool samples for the two ultradeep WMS sequencing analyses were recruited as part of research protocol number 150275 approved by the University of California San Diego Institutional Review Board. Research was conducted in accordance with the Helsinki Declaration. Informed consent was obtained from all subjects recruited into the study.

### Data availability.

The data for the ultradeep WMS sequencing have been deposited in the European Nucleotide Archive with the accession code PRJEB24152.
