# Supplementary figures and images for: Evaluating the Information Content of Shallow Shotgun Metagenomics
Source: mSystems. 2018 Nov 13;3(6):e00069-18. doi: 10.1128/mSystems.00069-18 (PMC6234283; doi:10.1128/mSystems.00069-18)

**A**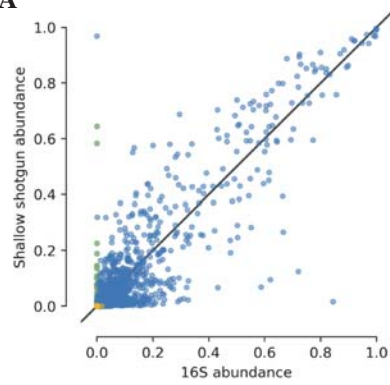**B**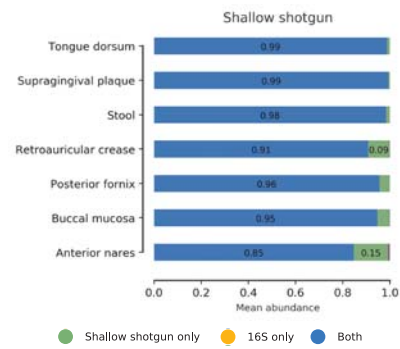**C**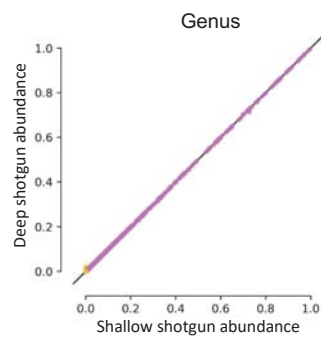**D**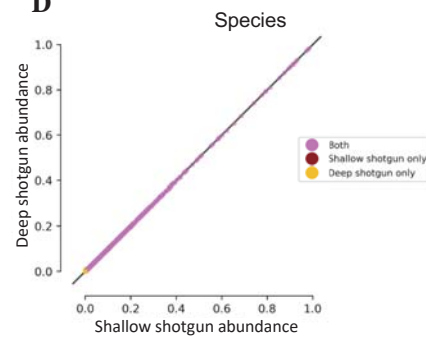

Supplement: FIG S1 [file sys005182277sf1.pdf]
